# Designing and Piloting a Program to Provide Water Filters and Improved Cookstoves in Rwanda

**DOI:** 10.1371/journal.pone.0092403

**Published:** 2014-03-27

**Authors:** Christina K. Barstow, Fidele Ngabo, Ghislaine Rosa, Fiona Majorin, Sophie Boisson, Thomas Clasen, Evan A. Thomas

**Affiliations:** 1 Civil, Environmental and Architectural Engineering, University of Colorado, Boulder, Colorado, United States of America; 2 Maternal and Child Health, Ministry of Health, Kigali, Republic of Rwanda; 3 Department of Infectious and Tropical Diseases, London School of Hygiene and Tropical Medicine, London, United Kingdom; 4 Department of Environmental Health, Rollins School of Public Health, Emory University, Atlanta, Georgia, United States of America; 5 Mechanical and Materials Engineering, Portland State University, Portland, Oregon, United States of America; University of Washington, United States of America

## Abstract

**Background:**

In environmental health interventions addressing water and indoor air quality, multiple determinants contribute to adoption. These may include technology selection, technology distribution and education methods, community engagement with behavior change, and duration and magnitude of implementer engagement. In Rwanda, while the country has the fastest annual reduction in child mortality in the world, the population is still exposed to a disease burden associated with environmental health challenges. Rwanda relies both on direct donor funding and coordination of programs managed by international non-profits and health sector businesses working on these challenges.

**Methods and Findings:**

This paper describes the design, implementation and outcomes of a pilot program in 1,943 households across 15 villages in the western province of Rwanda to distribute and monitor the use of household water filters and improved cookstoves. Three key program design criteria include a.) an investment in behavior change messaging and monitoring through community health workers, b.) free distributions to encourage community-wide engagement, and c.) a private-public partnership incentivized by a business model designed to encourage “pay for performance”. Over a 5-month period of rigorous monitoring, reported uptake was maintained at greater than 90% for both technologies, although exclusive use of the stove was reported in only 28.5% of households and reported water volume was 1.27 liters per person per day. On-going qualitative monitoring suggest maintenance of comparable adoption rates through at least 16 months after the intervention.

**Conclusion:**

High uptake and sustained adoption of a water filter and improved cookstove was measured over a five-month period with indications of continued comparable adoption 16 months after the intervention. The design attributes applied by the implementers may be sufficient in a longer term. In particular, sustained and comprehensive engagement by the program implementer is enabled by a pay-for-performance business model that rewards sustained behavior change.

## Introduction

Access to improved drinking water and clean burning stoves could benefit the millions who suffer from diarrheal disease and pneumonia, two of the leading causes of death around the world for children under five. Worldwide, of the 7.6 million deaths in children under 5 in 2010, 64% were associated with infectious diseases including 18% with pneumonia and 11% with diarrhea. Combined, pneumonia and diarrhea kill over 2 million children each year [1].

Some of these deaths may be avoided through interventions to improve indoor air quality and household water quality: pneumonia is often linked to indoor air pollution from biomass fuels [2], [3] and diarrhea to deficiencies in water and sanitation, including poor water quality [4]. Many cookstove interventions have shown a reduction in indoor air pollutants such as carbon monoxide and fine particulate matter [5], [6]. Similarly, interventions targeted at improving household water quality through the implementation of water treatment strategies such as chemical treatment, boiling, solar disinfection or filtration have been shown to reduce diarrheal disease [7], [8].

Even with the fastest annual reduction in child mortality in the world, the Republic of Rwanda still faces challenges related to pneumonia and diarrhea: among deaths of children under 5, pneumonia accounts for 18% and diarrhea for 8% [9]. Cooking practices in a rural Rwandan household may contribute to this pneumonia burden since the predominate fuel and cookstove pairing is wood on a three stone fire [10]. Additionally, while Rwanda has demonstrated significant progress towards the Millennium Development Goals, almost 30% of households do not have access to an improved water source [11], and the improved water sources may become contaminated during collection, transport or storage within the home [12], [13]. Once water is in the households, less than half (46.1%) of rural Rwandan families report treating their drinking water, with boiling as the leading treatment method (38.1%) [11], which again can become recontaminated after treatment [14]. The Rwanda Standard for Potable Water states that the microbiological limits for potable water for total CFU/100 ml of total coliforms should be 0 [15]. A baseline water quality assessment of 230 improved water sources, 78 unimproved water sources, and stored water in 468 households across all 30 districts in Rwanda indicated that 27.8% of improved water sources, 80.2% of unimproved water sources, and 58.3% of stored household water supplies exceed this standard [16], falling into the “intermediate”, “high” or “very high” risk World Health Organization categories [17] for biological contamination of drinking water supplies. Another study of households within the other 11 districts of the project area prior to the start of the program implementation indicated that 81.1% of households exceed this standard, with 59.1% falling into the “intermediate” or “high” risk categories [18].

DelAgua Health, a for-profit social enterprise, was established to combine household technologies that address environmental health issues with market-based mechanisms. DelAgua Health participates in the United Nations Clean Development Mechanism (CDM) to earn carbon credits associated with the reduced use of, and demand for, fuel wood associated with water treatment and cooking, and then sell those credits to buyers as a way to recover costs and profit [19].

Carbon finance markets facilitate the reduction of greenhouse gas emissions worldwide through economic incentives, while allowing cleaner economic development to take place. Each emission reduction credit represents the non-emission of one tonne of carbon dioxide into the atmosphere. The carbon credits generated under the CDM help Kyoto Protocol Annex I countries to meet their binding targets, and can be traded in the marketplace. However, the carbon markets have yet to be well utilized to finance the distribution of humanitarian technologies in the least developed countries, particularly in Africa. Although the CDM is a multi-billion dollar industry, fewer than 2 percent of projects are registered in African nations [20].

Depending on the project location, structure, methodology and registration mechanism employed, a water treatment and/or cookstove program can earn between approximately 1/2 and 5 carbon credits per household, per year. The carbon credits earned are a function of the approved methodology, referenced to a baseline condition and the current performance of the program, as audited by independent firms. The reported reductions are then issued by the registration authority and are then sold to buyers. Carbon credit buyers may be banks, energy companies, brokers, or sovereign nations who require credits for either regulatory compliance or voluntary social responsibility efforts, or both. Because the carbon credits are issued in proportion to the present adoption and proper use of intervention technologies, this encourages sustained engagement by the program implementer and creates a pay-for-performance model.

In Rwanda, DelAgua Health is partnered with the Ministry of Health since 2012 to distribute free of charge household water treatment and high efficiency cookstoves to approximately 600,000 households (about 3 million people), throughout the country’s 30 districts. The project will target *Ubudehe* categories 1 and 2, the government-recognized poorest 30% of the country. *Ubudehe* category is determined by community members based on classifications outlined by the Rwandan Ministry of Local Government [21]. Households categorized as *Ubudehe* 1 and 2 already receive free medical and other assistance through government programs.

A pilot program was initiated in October 2012 to provide input for the full effort, scheduled to start in mid-2014. This pilot was conducted after findings from a preliminary study of 100 households in July 2012. This effort was judged by the implementers to be sufficiently promising for testing at a larger scale. This paper discusses the design, development, implementation, monitoring and periodic modification of the October 2012 pilot program. We summarize the results of surveys collected to evaluate key outputs including intervention uptake and use. Other aspects of the pilot are described elsewhere, including a novel method for assessing intervention use with remotely reporting sensors [22] and a randomized controlled trial to study the impact of the intervention on drinking water quality and household air pollution [23].

## Materials and Methods

### Design Objectives

The objective of this study was to identify if certain design criteria, integrated together and applied to environmental health technologies, could result in a meaningful proportion of continued use of stoves and water filters. These design attributes are evaluated as a whole, though estimates of relative value are provided in the discussion. The three program design choices considered fundamental were:

1. **Free Distribution.** Free provisioning of high quality stoves and water filters under the authority of the Government of Rwanda and through established community mechanisms including community meetings and community leadership.
2. **Behaviour Change.** A behaviour change messaging and monitoring effort that prioritizes consistent and correct adoption of the stoves and filters through community and household level activities, focusing on both health and non-health benefits.
3. **“Pay for Performance” Public-Private Partnership**. A public-private partnership with the Rwanda Ministry of Health enabled by anticipated carbon credit revenues, which allows sustained, comprehensive community engagement by virtue of future anticipated “pay for performance” carbon credit revenues.

## Program Setting and Population

**Figure 1:**
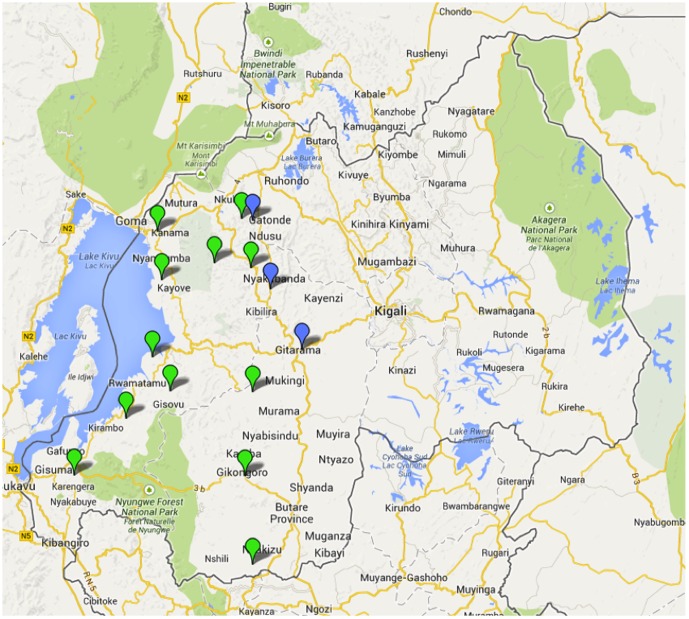
Program villages. RCT villages shown with blue pins.

The pilot was conducted in a convenience sample of 15 non-randomly selected villages spread across 11 districts in Western Rwanda (Figure 1). The 15 villages were selected to have at least one village per the 11 districts and the remaining four villages in districts with the largest populations. Additional inclusion criteria included ensuring that no villages were in adjacent sectors (district subdivisions), less than 20% of households in each village served by piped water; less than 60% of households in each village using any water treatment other than boiling; less than 20% of households in each village using cooking fuel sources other than biomass or charcoal; and less than 20% of households in each village using any stove other than a 3-stone fire or two other locally made unimproved stoves (known as *Rondereza* and *Imbabura* stoves). These inclusion criteria were selected by program staff to be representative of typical rural villages in Rwanda, based on rural water service and energy use characteristics identified in the Rwanda 2011 Demographic and Health Survey. Program staff visited each candidate village in advance to confirm with village officials that it met eligibility criteria. All 1,943 households who were registered as members of the 15 villages were eligible to participate in the study. While the full program will consist of distribution to only *Ubudehe* 1 and 2 households, this pilot program consisted of all households, of any *Ubudehe* category, in the 15 villages. The full program originally consisted of distribution to all households in the Western province of Rwanda but was later revised to be a country wide program of *Ubudehe* 1 and 2 households. This program change was directed by the Government of Rwanda Ministry of Health.

### Intervention Hardware

The water filter used in this program, the Vestergaard Frandsen LifeStraw Family 2.0 is a point-of-use microbial water treatment system intended for routine use in low-income settings. The system is a table-top unit where the user pours untreated water through a 20 micron pre-filter into a six liter influent water tank. Water is then gravity-filtered through a 0.20 micron hollow-fiber ultrafiltration membrane into a 5.5 liter safe storage container. Water can be dispensed from the safe storage container through a plastic tap, limiting recontamination. The filter is backwashed by squeezing a plastic bulb located on the opposite side of the tap. The membrane can filter up to 18,000 liters of water [24], enough to supply a family of five with microbiologically clean drinking water for three to five years. The system exceeds the ‘highly protective’ World Health Organization Standard for household water treatment technologies [25], [26]. In a recent study, an earlier version of this filter was shown to be highly effective in improving water quality and was protective against diarrhea among HIV positive individuals, reducing longitudinal prevalence by over 50% [27].

The cookstove used in this program, the EcoZoom Dura, is based on the rocket-stove concept that is designed to concentrate the combustion process while channeling air flow to create a more complete burn. A complete burn of carbon rich material will also result in little to no smoke. Included with the stove are a “stick support” on which fuelwood is placed to promote air flow and a “pot skirt” which increases thermal efficiency. In the field, performance is variable but when properly used, a rocket stove will significantly reduce fuelwood use by at least 50%, although reductions in indoor air pollution vary between designs, fuel types and use [28]. The thermal efficiency of this stove is 38% [29].

### Intervention Design

The program is designed leveraging established behavior change theories, including the Diffusion of Innovation theory [30] and the Health Belief Model (HBM) [31]. In particular, the program design assumes that continued, comprehensive engagement is critical in order to effect positive behavior change. The program design takes a hybrid approach, integrating pieces of these theories that apply in a Rwandan context to shape the communication strategy of the program.

Several components of the diffusion of innovation theory are applied to the program. At the initial distribution meeting, community members are informed about the potential health and other benefits of the water filter and cookstove creating ‘initial knowledge’ around the technologies. The ‘persuasion stage’ is initiated through demonstrations at both the community meeting and the household. The stove demonstration includes assembling the stove, how to adjust a pot skirt which can be fitted to different sized pots and finally a fire being started in the stove with some demonstrations including boiling a pot of water to show the rapidity of the cooking process. The water filter includes a demonstration of filtering visually dirty water with clear water coming out of the tap and the maintenance procedure, which includes backwashing the filter. Progressing to the ‘decision stage’ the household is then asked to demonstrate use and maintenance of the technology, allowing them to trial the technology. Households then move into the ‘implementation stage’ where they can choose to adopt or reject the technology. About a month later, the program implements the ‘confirmation stage’ where households who have chosen to partially adopt or reject the technology are given additional training and messaging to hopefully reverse their decision.

Village chiefs are promoted as ‘early adopters’ because of their high degree of influence and respect within their villages. In this program the change agents are Community Health Workers (CHWs). The CHW system in Rwanda includes three CHWs per village who are part-time volunteers of sector health centers and are compensated with a stipend. They provide basic services such as maternal and newborn health monitoring, vaccination advocacy, family planning, treatment of malaria, and sanitation and hygiene education. Through this program CHWs play several important roles including informing households of the need for the devices, encouraging adoption and analyzing potential problems with the technologies. The CHWs play an especially important role with the ‘late adopters’ and ‘laggards’ as more effort is needed to change the household’s old habit and promote the new behaviors [32].

The Health Belief Model is used to shape messaging. The belief that there is a health threat is compelled by messaging related to clean drinking water and clean indoor air. Households are educated about the reduced risk of diarrheal disease from water borne diseases and the reduced risk of respiratory problems from breathing indoor air pollutants. Additionally an important concept in social cognitive theory is often added to the health belief model, self-efficacy, which states that the user must believe that they can adopt the new behavior [33]. This is facilitated through households gaining confidence in the use of the technologies by having members of the household demonstrate proper use.

While the health belief model provides relevant guidance on behavior change theory it is important that the program also express non-health benefits to users. Previous interventions related to both water quality and improved cookstoves emphasize the need to highlight non-health benefits such as those related to economic and social benefits [32], [34]. Thus CHWs educate households on additional benefits such as reduction in medical costs from the water filter and a reduction in cooking time and expenditure on fuel costs for the cookstove.

### Adoption and Monitoring Survey

Households were assigned to receive the adoption and monitoring survey in one of six rounds by a random number generator. Approximately 325 households were surveyed each month with the exception of month five where approximately twice as many households were surveyed as rounds 5 and 6 were combined because of time constraints. Environmental Health Officers (EHOs) were responsible for conducting the surveys. Environmental Health Officers (EHOs) are full time employees of the Ministry of Health at sector health centers who are responsible for a range of health interventions, including, food safety, waste management, water, sanitation and hygiene inspection, indoor air pollution and environmental emergency health interventions [21]. Each household was surveyed by EHOs only once during the five month period.

The survey consisted of about 100 questions and was administered using a smartphone in Kinyarwanda by an EHO. Information included household identifying information, demographics, cooking practices, and water treatment and collection practices. The survey included both self-reported use questions to be answered by members of households and observational questions which EHOs answered based on their observations. Observational use of the water filter was measured by checking if water was present in the filter at the time of the visit while observational use of the stove was only confirmed if the stove was actually being used at the time of the visit.

### Survey Data Analysis

All survey data was uploaded to the doForms database where it was analyzed using T-SQL and R-Project. Only surveys that fell within 15 to 90 minute survey duration were included in the analysis. All numerical outcomes were additionally analyzed using an outlier analysis where only 1.5 times the upper and lower interquartile range were included in that particular outcome. This outlier exclusion was chosen to be consistent with the program’s carbon credit monitoring requirements [35][. Analysis of variance was used to compare group means. Additionally any missing data was excluded from the analysis.

### Focus Group Discussions

Three focus group meetings were conducted concurrently with EHOs and CHWs to assess qualitative aspects of the program. A total of 30 participants attended the meetings including one CHW from each of the pilot villages and all EHOs. CHWs were chosen by DelAgua staff as the highest performing CHWs within each village. Topics covered included perceived adoption of technologies within their villages, problems with filter and stove hardware, effectiveness of household messaging and boundaries to exclusive adoption of the filter and stove.

### Ethics and Consent

The study was reviewed and approved by the Rwanda National Ethics Committee (IRB #328/RNEC/2012), University of Colorado Institutional Review Board (Protocol #12-0564), and the London School of Hygiene and Tropical Medicine Ethics Committee (Trial registration: Clinicaltrials.gov NCT01882777). Each participating household gave informed, verbal consent after having received complete details regarding the purpose of the survey as well as information regarding privacy of personal information. Enumerators were required to confirm electronically, on their smartphone surveys, if a.) the respondent was over 18, and b.) if they gave verbal consent, before the smartphone allowed the survey to continue. These consent records are kept on a password protected server. Verbal consent was requested and approved by the approving ethics committees, based on the high percentage of illiteracy within the study population. Rwandan residents are often asked about their water and energy habits by community health workers, and the signing of a document adds a level or formality that may mislead participants. Participants were given the opportunity to ask any questions before agreeing to participate. All households were entitled to retain their filters and stoves at the conclusion of the study.Participation in the study was not a prerequisite to receiving the filters and stoves.

## Results

### Program Delivery

All households that were registered in the 15 villages according to the village chief’s list were distributed a stove and filter. The model of distributing at a central location allowed for the implementer to transport the technologies to a location that could be reached by vehicles of which many households could not. It was then members of the household’s responsibility to get the stove and filter to their homes.

### Household Characteristics

A total of 1943 households participated in the study, from which 1634 (84.1%) valid surveys were included in the analysis. Selected household characteristics are summarized in Table 1. The mean household size was 4.55, consistent with Rwanda’s 2011 Demographic and Health Survey household size of 4.5. Approximately one-third (29.3%) of households reported being categorized as *Ubudehe* 1 or 2, 70.5% were classified as 3 or 4 and 0.2% as *Ubudehe* 5 or 6. *Ubudehe* 1 and 2 household size was significantly lower than the entire study population with 3.85 persons per household. However, fuel type and water source were similar with the majority of all households (92.4%) and *Ubudehe* 1 and 2 households (89.1%) using wood as their primary fuel source and all households (41.1%) and *Ubudehe* 1 and 2 households (42.9%) reporting a public tap as their primary drinking water source.

**Table 1:** Selected demographics and characteristics regarding water and energy practices.

	All households		*Ubudehe* 1 and 2	
	N	%	N	%
**Number of Households**	1634		478	
Household size, mean (95%CI)	4.55 (4.46–4.65)		3.85 (3.70–4.00)	
***Ubudehe*** ** Category**				
1 or 2	478	29.3%		
3 or 4	1152	70.5%		
5 or 6	4	0.2%		
**Fuel Type**				
Wood	1510	92.4%	426	89.1%
Straw/Shrubs/Grass	93	6.3%	42	8.8%
Charcoal	28	1.9%	10	2.1%
LPG/Natural Gas/Biogas	1	0.1%	0	0.0%
Other	2	0.1%	0	0.0%
**Drinking water source** *	1576		457	
Public Tap	647	41.1%	196	42.9%
Protected Spring	592	37.6%	149	32.6%
Unprotected Spring	184	11.7%	62	13.6%
Surface Water	56	3.6%	26	5.7%
Hand Pump	37	2.3%	6	1.3%
Piped Water in Home	31	2.0%	8	1.8%
Unprotected Well	24	1.5%	8	1.8%
Protected Well	4	0.3%	1	0.2%
Rainwater	1	0.1%	1	0.2%

*Missing 58 (3.6%) answers.

### Filter Adoption and Use

Adoption of the LifeStraw filter was measured at approximately 90% or greater by several metrics. Households reported use of the filter had the highest adoption rate with 96.5% of households reporting the water filter as the treatment method for the last water they drank. An observational measure of use through presence of water in the filter showed a slightly lower adoption rate with 9 out of 10 households having water in their filter at the time of household visit (Table 2). Similar adoption rates as measured observationally were seen over the five follow up visits with the first follow up visit having the highest adoption rate of 92.6% and the lowest adoption rate reported as 86.4%. No longitudinal trend in adoption was observed through the five months of the study.

**Table 2:** Primary filter adoption and use outcomes.

	All households				*Ubudehe* 1 and 2			
	N	%	Mean (95% CI)	ANOVA p-value	N	%	Mean (95% CI)	ANOVA p-value
**Reported Filter Use**	1576	96.5%			457	95.6%		
**Observed Filter Use**	1471	90.0%			423	88.5%		
**Other Purposes for Filtered Water**	201	12.8%			74			
Cooking	57	28.4%			24	32.4%		
Hand Washing	57	28.4%			20	27.0%		
Washing Dishes	55	27.4%			18	24.3%		
Other	32	15.9%			12	16.2%		
**Cleaning Frequency**	1576				457			
Everytime	1004	63.7%			265	58.0%		
Daily	234	14.8%			71	15.5%		
Every Other Day	53	3.4%			20	4.4%		
2 Times per Week	236	15.0%			83	18.2%		
Once per Week	48	3.0%			18	3.9%		
Once per Month	1	0.1%			0	0.0%		
**Liters per household per day**	1210*		5.06 (4.99–5.12)	0.193	304*		5.11 (5.04–5.18)	0.194
**Liters per person per day**	1436*		1.27 (1.25–1.29)	0.090	304*		1.11 (1.10–1.13)	0.194

*Only records within 1.5 IQR were included.

Adoption rates were further analyzed to understand any differences between all households in the study and those who were identified as *Ubudehe* 1 and 2 as well as any differences across the 15 villages. *Ubudehe* 1 and 2 adoption rates were similar to those seen by the full program with an *Ubudehe* 1 and 2 reported adoption rate of 95.6% and an observational adoption rate of 88.5% (Table 2). Observed filter adoption across all 15 villages varied between 74.5% and 98.1% (Table 3) with the lowest adoption rate occurring in one village (Mara) by almost 10% below all other villages. This is likely due to rodents destroying the filter tubes with 21.8% of filter repair of this problem in this village.

**Table 3:** Filter and stove use by village.

	Observed Filter Use			Cooking on EcoZoom only at time of visit		
Village	Valid observations	N	%	Valid observations	N	%
Mara	110	82	74.5%	35	24	68.6%
Kigaga	90	82	91.1%	23	19	82.6%
Buhunde	117	103	88.0%	31	11	35.5%
Rushishi	147	137	93.2%	26	15	57.7%
Nyarubuye	116	102	87.9%	16	10	62.5%
Karambo	177	149	84.2%	34	23	67.6%
Rubona	65	62	95.4%	21	13	61.9%
Burorero	214	191	89.3%	18	7	38.9%
Nyabivumu	61	58	95.1%	22	22	100.0%
Rambura	116	107	92.2%	12	6	50.0%
Kabuga	111	106	95.5%	23	9	39.1%
Rupango	106	102	96.2%	31	27	87.1%
Gisoro	54	53	98.1%	8	3	37.5%
Nyarutovu	81	77	95.1%	31	21	67.7%
Gasumo	69	60	87.0%	11	8	72.7%
All villages	1634	1471	90.0%	342	218	63.7%

Households reported treating an average of 5.06 liters per day in all households and 5.11 liters per day in *Ubudehe* 1 and 2 households with no significant difference between monthly survey rounds. This equates to an average of 1.27 liters per person per day for all households and 1.11 liters per person for *Ubudehe* 1 and 2 households, possibly because of the smaller household sizes (Table 2). Regardless, water quantity consumption is lower than advised by CHWs at 2 liters per person per day. Similar consumption rates of 1 to 1.5 liters per person per day were reported at focus group discussions conducted with community health workers. Primary reasons discussed at the focus group for not consuming more water included not having a container to carry water when leaving the household, an inability to drink 2 liters per day, and a preference for drinking other beverages.

12.8% of households that reported using the filter also reported doing so for purposes other than drinking water. The most common uses were cooking (28.4%), hand washing (28.4%) and washing dishes (27.4%) (Table 2).

Almost two thirds (63.7%) of households reported backwashing their filter every time they treated water as advised in the household visit (Table 2). Not backwashing the filter frequently enough may be the cause of the most common reported problem with the filter, which was that it was clogged and wouldn’t filter water (N = 45). The next most common problem reported was damage to rubber tubes because of rodents (N = 33). Overall 11.1% of households over the 5-month period reported any problems with the filter during the household visits. 57 (2.9%) filters were replaced and 366 (18.8%) filters were repaired with the same primary reported problems of tubes being damaged by rodents (N = 119) and filter clogged (N = 124) (Table 4).

**Table 4:** Reasons for stove and filter problems, repairs and replacements.

	N	%
**Reported reasons for not using EcoZoom**	168	
Don't have dry wood	46	27.4%
Difficult to use	31	18.5%
Doesn't warm the house	20	11.9%
Use of a different fuel	13	7.7%
Other (20)	76	45.2%
**Reported stove problems**	73	
Pot skirt missing screw	22	30.1%
Pot skirt damaged	8	11.0%
Stove too small	6	8.2%
Difficulty in moving pot skirt to another pot	6	8.2%
Ceramic chamber cracked	4	5.5%
Stick support damaged	4	5.5%
Other (12)	23	31.5%
**Reported reasons for continued use of old stove**	1386	
More than one stove needed	649	46.8%
Don't have dry wood	330	23.8%
Need to warm house	126	9.1%
Pot is too big for stove	73	5.3%
Other (28)	208	15.0%
**Reasons for stove repair**	67	
Skirt replaced/Skirt damaged	48	71.6%
Skirt replaced/adjustment screws missing	11	16.4%
Stick support replaced/Broken	3	4.5%
Stick support replaced/Missing	1	1.5%
Other	4	6.0%
**Reported filter problems**	182	
Filter broken or clogged	45	24.7%
Tubes damaged/eaten by rodents	33	18.1%
Tubes are kinked	26	14.3%
Difficulty in backwashing	22	12.1%
Tap is leaking or broken	18	9.9%
Backwash bulb is damaged	17	9.3%
Other (6)	21	11.5%
**Reasons for filter repair** *	391	
Filter cartridge clogged	124	31.7%
Tubes replaced from rodent damage	119	30.4%
Backwash water not going into container	62	15.9%
Broken tap handle	39	10.0%
Broken tap - leaking	10	2.6%
Backwash leaking	5	1.3%
Backwash bulb replaced	2	0.5%
Other	30	7.7%

*366 total filters repaired - 25 had multiple problems.

The majority of filter problems were addressed through repair and replacement by program staff. Households contacted program staff through phone numbers on informational posters which were provided during the initial household visits. Common repairs included replacing tubes eaten by rodents or power backwashing the filters using a hand pump pressurized canister.

### Stove Adoption and Use

As seen with filter adoption, reported primary use of the EcoZoom stove was around 90% for the entire population and *Ubudehe* 1 and 2 households (Table 5). Primary reasons given for stove adoption during focus group meetings included cost savings, time savings and cleanliness of the cook and kitchen when using the EcoZoom. However, 71.5% of these households reported continuing to use their traditional stove as well as their EcoZoom stove. Of households cooking at the time of the follow up visit (20.9%), about two thirds (63.7%) were using the EcoZoom stove, 21.9% a traditional 3-stone fire, and 11.4% cooking on a different traditional stove. Ten households (2.9%) were also observed using both the EcoZoom stove and a traditional stove.

**Table 5:** Primary stove adoption and use outcomes.

	All households				*Ubudehe* 1 and 2			
	N	%	Mean (95% CI)	ANOVA p-value	N	%	Mean (95% CI)	ANOVA p-value
**Reported Primary Stove Use**	1634				478			
EcoZoom	1479	90.5%			430	90.0%		
Traditional 3-stone Fire	68	4.2%			23	4.8%		
Other Traditional Stove	78	4.8%			22	4.6%		
Other Stove	9	0.6%			3	0.6%		
**Stove Using at Time of Visit**	342	20.9%			110	23.0%		
EcoZoom Only	218	63.7%			70	63.6%		
Traditional 3-Stone Fire	75	21.9%			28	25.5%		
Other Tradional Stove	39	11.4%			11	10.0%		
EcoZoom and Other Stove	10	2.9%			1	0.9%		
**Cooking Location at Time of Visit**								
Outdoor	145	42.4%			41	37.3%		
Indoor	123	36.0%			49	44.5%		
Separate Kitchen	74	21.6%			20	18.2%		
**Continuing to Use Old Stove**	1169	71.5%			316	66.1%		
**# of EcoZoom uses per week**	1517*		1.37 (1.34–1.40)	<0.000001	438*		1.38 (1.32–1.44)	0.869
**% EcoZoom Use**	1625*		71.2% (69.8%–72.7%)	0.052	474*		73.7% (71.0%–76.4%)	0.896
**% Wood Reduction**	1551*		65.8% (65.0%–66.6%)	0.593	443*		65.9% (64.3%–67.4%)	0.082

*Only records within 1.5 IQR were included.

Using the same metric of observed cooking use, EcoZoom use at the time of the household visit varied from 35.5% – 100.0% across the 15 pilot villages though the sample sizes were low in some villages with only 342 total observed cooking events. The three villages with the lowest observed use were Buhunde, Gisoro and Burorero (Table 3).

The two primary reasons reported during household surveying for not using the EcoZoom stove were inability to use wet wood in the EcoZoom stove (N = 46) and difficulty in using the stove (N = 31). This reported difficulty may refer to the required increased frequency of fire tending while cooking on the EcoZoom stove as 67.1% of households reporting tending the fire more with the EcoZoom stove than with their old stove (Table 4). Additionally the food most frequently reported cooked was beans (53.9%), requiring cooks to tend the fire frequently over a long period of time. Focus group discussions further emphasized these problems with the primary reasons for not using the stove as high frequency of fire tending, difficulty in burning wet wood when dry wood was unavailable and the inability to warm the house. Tending the fire was expressed most frequently as the primary issue since cooks use smaller pieces of wood to keep the fire going.

To assess the degree of EcoZoom stove use compared to other traditional stoves, households were asked the number of times per week they used each stove in their home. The EcoZoom stove was reported being used on average 1.37 times per day for 71.2% of cooking events in a household with a significant difference between monthly survey rounds (Table 5).

Primary reasons for continued use of a traditional stove included needing more than one stove at a time (N = 649) and the inability to cook on the EcoZoom stove when only wet wood was available (N = 330) (Table 4).

Cooking location was assessed because of the program emphasis on cooking outdoors during education and training activities. 342 observations of cooking location at the time of follow up visit were made, where 42.4% were cooking outdoors, 36.0% were cooking indoors and 21.6% in a separate kitchen. Slightly higher rates of cooking indoors were observed in the *Ubudehe* 1 and 2 households with 37.3% cooking outdoors, 44.5% cooking indoors, and 18.2% cooking in a separate kitchen (Table 5).

To quantify wood savings households were asked to report the number of wood bundles they collected or purchased before and after receiving the EcoZoom stove. Of the 1551 valid responses, an average wood reduction of 65.8% was reported across all rounds with no significant difference between the five rounds (Table 5).

A total of 73 stove problems were reported with the two most common problems being the pot skirt screw missing (N = 22) and the pot skirt degrading (N = 8). These were also the two most common reasons for stove repair with 48 pot skirts (2.5%) being replaced due to melting and 11 pot skirt replacements due to missing adjustment screws (Table 4). No stoves were replaced during the five months following distribution.

## Discussion and Conclusions

In the pilot program described here high levels of uptake and continued use of water filters and improved cookstoves were found. The rigorous five-month follow up study was complemented by qualitative assessments by the implementation team periodically over an additional 11 months. 16 months after the intervention, adoption rates of the water filters and cookstoves were assessed to being comparable to those observed during the detailed 5-month study. This outcome may be described through the three design choices outlined previously. These design choices are intended to be applied in the full-scale program scheduled for deployment in 2014 and 2015. A key design difference in the planned full-scale program is that it will reach only the poorest households in a given village.

### Behavior Change

The primary purpose of the technologies provided is to realize a health benefit. As a first step, communicating these potential health benefits to a user is often seen as an appropriate prerequisite to adoption. A lack of knowledge of potential health benefits has been shown to result in poor adoption of products like stoves and filters [36]. It has also been demonstrated that knowledge of health benefits alone is not sufficient to result in sustained behavior change in an individual or household [34]. The program studied here uses theories of behavior change such as diffusion of innovation and the health based model with both health based messaging as well as economic and social messaging to promote behavior change within the program.

In the case of the filter, adoption was measured around 90% for the five months of the study. A high adoption outcome has been seen previously with earlier versions of the LifeStraw Family filter with 96% adoption in Zambia [27] after 12 months and 68% adoption in the Democratic Republic of Congo after eight months [37]. Additionally, compared to other point-of-use water methods, filtration often has higher adoption rates [38] possibly because it is seen as easier to use [39] and doesn’t result in a change in taste and odor [34]. However, while adoption of the filter was high, the recommended water consumption of 2 liters per person per day was not reached in most cases, suggesting that households may be drinking untreated water at times, a behavior also seen in the study conducted in the Democratic Republic of Congo [37]. Even occasional consumption of untreated water can greatly reduce the potential health benefits from water quality interventions [40], [41]. Reasons that households gave for not exclusively drinking clean water included not having clean water when they were away from home and not having any filtered water at the time they were thirsty. Similar reasoning has been found in other studies where water treatment needed to be integrated into the everyday lifestyle of the family [34]. However, guidance from the existing literature is limited to help guide behavior change program development [42] so additional qualitative research is needed to understand people’s behavior and preferences for exclusive drinking of treated water to adjust messaging and education activities to be more effective. In collaboration with the manufacturer, the product has been updated based on recipient feedback to protect the soft tubes and the backwash bulb, and to allow for a separable safe water storage container for ease of cleaning.

Adoption of the stove was also around 90%. Non-health benefits such as a cleaner appearance and cooking environment were more highly valued than health or environmental impacts, as observed in other studies. Exclusive use of the EcoZoom stove was only reported in 28.5% of households with most continuing to use their old stove with the EcoZoom stove. The earliest models for stove adoption assumed a “fuel switch” wherein behavior is switched over a short period of time from one stove/fuel combination to another. More recently, continued “stove stacking”, where the use of multiple stoves for varying purposes, has been shown to be a more stable behavior, and can result in as high as 90% of stove usage events on the improved stove [43]. Studies have shown that households do not move from older existing methods of cooking such as a 3-stone fire to exclusive use of an improved stove. In order to realize the potential health benefits of improved stoves, exclusive use will need to be further promoted within the program [44], [45], [46]. Working with the stove manufacturer, the pot skirt has been updated to reduce degradation.

This stove stacking behavior suggests that the true innovation being introduced is not necessarily the stove itself, but the modified cooking practices required to realize the health and other benefits. Previous studies have found that an improved stove must meet the user’s traditional cooking needs in order for adoption to occur. The primary barriers to adoption or exclusive use of the EcoZoom stove in this study center around modifying current practices such as additional fire tending. While the EcoZoom stove is likely to decrease the cooking time of an individual cooking event, additional fire tending compared to a traditional 3-stone fire is often necessary, as reported in the focus group convened for this study. There may be an increased emissions exposure risk associated with greater fire tending that has not yet been characterized. When cooking meals on a 3-stone fire that requires long cooking times, such as beans, the cook will often prepare a large fire and perform other household tasks while the beans are cooking. Use of the EcoZoom stove requires a behavior modification to persuade the cook to stay by the fire while the meal is cooking. Additionally burning of wet wood in an improved stove can be more difficult than a 3-stone fire, so a cook often prefers to use the easier cooking method and therefore it is suggested that a careful examination of cooking practices, and focusing on those practices rather than the intrinsic benefits of the technology may result in higher adoption rates [43].

42.4% of observed cooking events occurred outdoors as instructed through education and training activities. While the EcoZoom stove has the potential to reduce indoor air pollution compared to traditional stoves, the primary reduction may likely come from moving cooking out of the home. As less than half (42.4%) of observed cooking events occurred outdoors as instructed through education and training activities, further messaging targeted at cooking location will need to be performed to increase outdoor usage.

A 65.8% reduction in wood usage by users of the EcoZoom stove is likely to significantly reduce time to collect wood and expenditure on fuelwood. Much of the wood use in Rwanda is with small sticks and branches which burn fast on three stone fires. Wood reduction was calculated through the ratio of wood bundles used before and after receiving the EcoZoom stove. In order to better quantify wood savings, additional methods will need to be employed to better understand wood usage such as the kitchen performance test which can evaluate stove performance in real-world settings [47].

With respect to both the filter and stove, behavior in *Ubudehe* categories 1 and 2 was similar to the overall population. This suggests that similar results could be expected during a large distribution of only *Ubudehe* 1 and 2 households. However, the effect of distributing to only a part of a village while the other households do not receive the technologies is unknown.

### Free Distribution

Recent studies have examined cost-sharing for bed-nets, cook-stoves and water treatment systems and have found that there is no correlation between free distributions and low adoption rates [48]. Meanwhile, a study examining point-of-use chlorination through marketing campaigns and coupon schemes found these to be ineffective strategies but found free chlorination dispensed at water sources along with community providers as the most effective strategy in potentially preventing diarrheal incidence in areas like rural Kenya [49]. Furthermore, Bensch and Peters determined that a free stove program in Senegal resulted in high uptake of almost 100% of households [50].

These studies suggest that adoption and price are not fundamentally correlated, and that other factors including community engagement, government support and education are worth more careful study. With respect to the private-public partnership described here, the free giveaway nature of the program did not appear to adversely affect technology adoption on a community level, and resulted in a broader population exposure to the interventions than would have been possible via a retail effort over the same time period. The high rate of exclusive use of filters suggest that free distribution did not impact filter use, though it may have impacted intervention stove use.

### Public-Private Partnership

The extensive logistical and behavior change messaging components of this program require sustained funding. Rwanda is not yet able to finance all health service activities directly; it relies both on direct donor funding to government programs, as well as careful coordination of programs managed by international non-profits and health sector businesses. By 2002, the government was spending 8.6% of its revenue on health care, which was only a third of the total costs, the remainder covered by donors [47]. Donation based non-profits are not providing services to the target populations serviced by this program. The business model anticipated by the for-profit implementer is designed to recuperate invested costs by the generation and sale of carbon credits associated with the proportion of the intervention that continues to demonstrate successful behavior change. The outcomes observed to-date support the business model in that high adoption rates will correlate to carbon credit generation sufficient to generate sustainable revenue that will allow continued program investment.

### Study Limitations

Many of the results described here are from self-reported survey data that may result in over-reporting because of courtesy bias [51]. Over-reporting was measured in this study through the use of remote sensors which revealed over-reporting in frequency of use of both the water filter and cookstove [22]. This contributes to existing evidence of courtesy bias in self-reported outcomes of product distributions. Additionally while the survey directly asked households about their use of other cookstoves, it did not ask about households about supplementing their drinking water from other sources. To fully understand this issue, additional surveying and analysis is necessary. Respondent fatigue may also have been an issue throughout the study as some households were visited several times during a single month. Additionally the short duration of this study (five months) with less rigorous follow up through at least month 16 doesn’t allow for complete characterization of the technologies or long-term adoption.
